# MethCORR infers gene expression from DNA methylation and allows molecular analysis of ten common cancer types using fresh-frozen and formalin-fixed paraffin-embedded tumor samples

**DOI:** 10.1186/s13148-021-01000-0

**Published:** 2021-01-28

**Authors:** Trine B. Mattesen, Claus L. Andersen, Jesper B. Bramsen

**Affiliations:** Department of Molecular Medicine, Aarhus University Hospital, Palle Juul-Jensens Boulevard 99, 8200 Aarhus N, Denmark

**Keywords:** Cancer, DNA methylation, Gene expression, RNA sequencing, FFPE tissue, Biomarkers, Molecular subtypes

## Abstract

**Background:**

Transcriptional analysis is widely used to study the molecular biology of cancer and hold great biomarker potential for clinical patient stratification. Yet, accurate transcriptional profiling requires RNA of a high quality, which often cannot be retrieved from formalin-fixed, paraffin-embedded (FFPE) tumor tissue that is routinely collected and archived in clinical departments. To overcome this roadblock to clinical testing, we previously developed MethCORR, a method that infers gene expression from DNA methylation data, which is robustly retrieved from FFPE tissue. MethCORR was originally developed for colorectal cancer and with this study, we aim to: (1) extend the MethCORR method to 10 additional cancer types and (2) to illustrate that the inferred gene expression is accurate and clinically informative.

**Results:**

Regression models to infer gene expression information from DNA methylation were developed for ten common cancer types using matched RNA sequencing and DNA methylation profiles (HumanMethylation450 BeadChip) from The Cancer Genome Atlas Project. Robust and accurate gene expression profiles were inferred for all cancer types: on average, the expression of 11,000 genes was modeled with good accuracy and an intra-sample correlation of *R*^2^ = 0.90 between inferred and measured gene expression was observed. Molecular pathway analysis and transcriptional subtyping were performed for breast, prostate, and lung cancer samples to illustrate the general usability of the inferred gene expression profiles: overall, a high correlation of *r* = 0.96 (Pearson) in pathway enrichment scores and a 76% correspondence in molecular subtype calls were observed when using measured and inferred gene expression as input. Finally, inferred expression from FFPE tissue correlated better with RNA sequencing data from matched fresh-frozen tissue than did RNA sequencing data from FFPE tissue (*P* < 0.0001; Wilcoxon rank-sum test).

**Conclusions:**

In all cancers investigated, MethCORR enabled DNA methylation-based transcriptional analysis, thus enabling future analysis of cancer in situations where high-quality DNA, but not RNA, is available. Here, we provide the framework and resources for MethCORR modeling of ten common cancer types, thereby widely expanding the possibilities for transcriptional studies of archival FFPE material.

## Background

In the clinic, patients are stratified based on staging of their tumors, which is founded in clinico-pathological evaluation of tumor appearance, growth pattern, and extend of disease spread. Despite this, cancers of similar stage still exhibit great differences in clinical outcome [1]. Probably because the inter-tumor heterogeneity at the molecular level still is very high within each tumor stage, causing “one-size fits all” treatment strategies to fail.

Recently, transcriptomic data have been widely used to resolve this molecular heterogeneity, e.g., by stratification of tumors into homogenous molecular subtypes [2–7] and application of subtype-specific biomarkers [5]. This paradigm-changing research was primarily performed using high-quality RNA purified from fresh-frozen tumor samples. Yet, fresh-frozen tumor tissue is not routinely collected in the clinic. Here, formalin-fixation and paraffin-embedding (FFPE) is the standard method for preservation and storage of tissue. As the quality of RNA purified from FFPE tissue is variable and often poor [8], transcriptional profiling of FFPE tissue samples can be challenging [9–11]. This currently complicates broad clinical testing of promising transcriptional biomarkers. Additionally, since FFPE preservation has been used for decades, large biobanks of archival FFPE samples with long-term clinical follow-up information exist. These represent a highly desirable resource for retrospective studies of tumor classification and to derive more focused biomarkers such as subtype-specific biomarkers [5, 12].

To unlock the potential of archival FFPE samples for molecular analysis and facilitate broad clinical testing, we previously developed the MethCORR method [12] using colorectal cancer (CRC) samples. MethCORR uses DNA methylation levels of RNA expression-correlated CpG sites, located anywhere in the genome, to infer RNA expression (iRNA) for a large fraction of genes using regression modeling (11,222 genes in CRC). As input, MethCORR utilizes DNA methylation profiles generated by the Illumina Infinium BeadChip platform (450K/EPIC), which can produce highly concordant DNA methylation profiles in matched fresh-frozen and FFPE samples [13–15]. In agreement, we have shown that MethCORR is compatible with both fresh-frozen and FFPE colorectal cancer tissue and that MethCORR allows uniform molecular characterization, classification, and prognostic biomarker identification independently of preservation type [12]. Based on these results, we hypothesized that the MethCORR method may be applicable to other cancer types as well and enable transcriptional analysis of samples with low RNA quality or when only DNA methylation profiles are available.

In this study, we demonstrate the applicability of the MethCORR method to ten other cancer types by exploiting the availability of matched RNA expression and DNA methylation data from The Cancer Genome Atlas project (TCGA), in order to identify RNA expression-correlated CpG sites in each cancer type. Our primary aim was to demonstrate the generality of the MethCORR method in cancer samples and to present MethCORR models that can be used to predict RNA expression from DNA methylation profiles in each cancer type. Secondarily, by focusing on breast, lung, and prostate cancer, we illustrated the potential use of inferred RNA expression profiles for molecular classification and characterization using fresh-frozen and FFPE cancer cohorts.

## Results

### MethCORR infers RNA expression from DNA methylation in ten cancer types

**Fig. 1:**
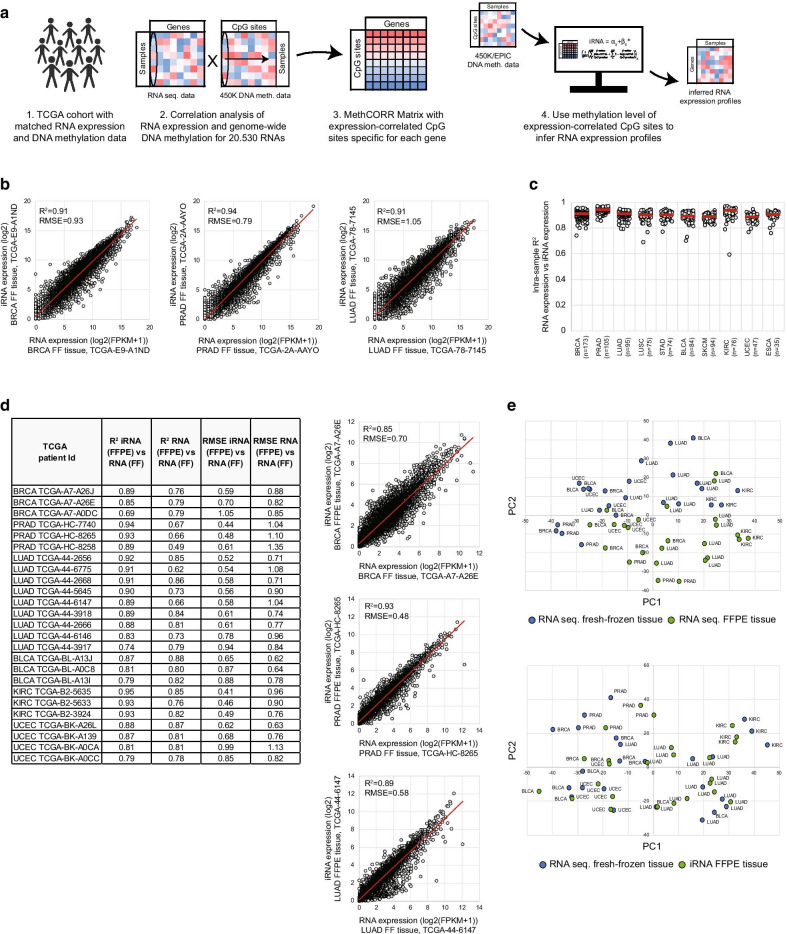
MethCORR inferred RNA expression in ten cancer types. **a** Overview of the MethCORR method. (1) Each TCGA cohort with matched RNA expression and DNA methylation data is independently used for the MethCORR method. (2) The expression of each RNA is correlated to the methylation level of each CpG site across all discovery samples. (3) The ≤ 100 most positive and ≤ 100 most negative expression-correlated CpG sites specific for each RNA constitute the MethCORR matrix. (4) The methylation level of the RNA expression-correlated CpG sites from the MethCORR matrix is used to calculate inferred RNA expression for each gene in fresh-frozen and FFPE samples. The iRNA expression profiles can be used for transcriptional-like analysis. **b** Scatterplots with intra-sample correlations between observed RNA expression and iRNA expression for a representative sample (median *R*^2^) from the TCGA BRCA, PRAD, and LUAD validation samples. **c** Scatterplot with RNA expression-iRNA expression squared correlations (*R*^2^) for all validation samples for each of the ten TCGA cohorts. **d** Left: Table with squared correlation (*R*^2^) and root mean square error (RMSE) for correlations between observed RNA expression in fresh-frozen tissue and iRNA expression calculated in matched FFPE tissue or observed RNA expression in matched FFPE tissue. Correlations are shown for all validation samples with matched tissue for the BRCA (*n* = 3), PRAD (*n* = 3), LUAD (*n* = 9), BLCA (*n* = 3), KIRC (*n* = 3), and UCEC (*n* = 4) cohorts. Right: Scatterplots with correlations between observed RNA expression in fresh-frozen tissue and iRNA expression calculated in matched FFPE tissue for a representative independent validation sample from the TCGA BRCA, PRAD, and LUAD cohorts. **e** Scatterplot with the first- (PC1; X-axis) and second principal component (PC2; Y-axis) from a PCA performed with RNA expression for 25 fresh-frozen cancer samples and matched FFPE RNA sequencing data (top) or calculated iRNA expression (bottom). The analysis was performed with common MethCORR genes for the 25 cancer samples (six cancers; *n* = 2374 common MethCORR genes)

The MethCORR method [12] was applied to data from ten cancer types (BRCA, PRAD, LUAD, LUSC, SKCM, STAD, BLCA, KIRC, ESCA, and UCEC; Table 1 and Additional file 1). In brief, this involved application of the following steps in samples used for training (80% of samples for each cancer; Fig. 1a): (1) identification of all genome-wide correlations between gene expression and CpG-site methylation levels using matched RNA sequencing and 450K methylation data. (2) Gene-wise selection of CpG sites (up to 200 sites per gene) whose methylation level most negatively- and positively correlated with expression (≤ 100 negatively and ≤ 100 sites positively correlated CpG sites). (3) Calculation of a MethCORR score (MCS) for each gene using the 200 expression-correlated CpG sites (see “Methods” section). (4) The MCSs were next used as input in linear regression modeling to identify genes for which the MCS can be used to infer RNA expression with good accuracy (as evaluated both by cross-validation and in completely independent samples; see methods section). A good relationship between observed RNA expression and inferred RNA expression (iRNA) was reached for 9313–13,018 genes, dependent on the cancer type analyzed (average of 11,000 genes; *R*^2^ > 0.16; Table 1; inter-sample modeling metrics can be found in Additional file 2). These genes, with high inter-sample correlations, were termed MethCORR genes. An investigation of all MethCORR models revealed that genes with good models (i.e., MethCORR genes) exhibited greater variation in RNA expression between samples as compared to genes with poor performing MethCORR models (non-MethCORR genes; *R*^2^ ≤ 0.16; Additional file 5: Fig. S1a), as previously described for colorectal cancer [12]. Hence, RNA expression variation between samples is needed for accurate MethCORR modeling and may explain why the number of MethCORR genes differ slightly between cancer types.

**Table 1:** Overview of cancer cohorts used in the study

Cohort	Cancer type	Number of samples	Available datatypes	Number of MethCORR genes
UCSC XENA TCGA BRCA	Breast invasive carcinoma	873	RNA seq 450K DNA meth	13,018
UCSC XENA TCGA PRAD	Prostate adenocarcinoma	533	RNA seq 450K DNA meth	11,348
UCSC XENA TCGA LUAD	Lung adenocarcinoma	477	RNA seq 450K DNA meth	11,935
UCSC XENA TCGA LUSC	Lung squamous cell carcinoma	379	RNA seq 450K DNA meth	10,911
UCSC XENA TCGA STAD	Stomach adenocarcinoma	372	RNA seq 450K DNA meth	11,259
UCSC XENA TCGA BLCA	Bladder urothelial carcinoma	424	RNA seq 450K DNA meth	11,238
UCSC XENA TCGA SKCM	Skin cutaneous melanoma	474	RNA seq 450K DNA meth	9473
UCSC XENA TCGA KIRC	Kidney renal clear cell carcinoma	343	RNA seq 450K DNA meth	10,725
UCSC XENA TCGA UCEC	Uterine corpus endometrial carcinoma	197	RNA seq 450K DNA meth	9313
UCSC XENA TCGA ESCA	Esophageal carcinoma	182	RNA seq 450K DNA meth	10,786
GSE117439	Breast cancer	52	450K DNA meth	–
GSE84207	Breast cancer	279	450K DNA meth	–
GSE73549	Prostate cancer	57	450K DNA meth	–
GSE66836	Lung adenocarcinoma	164	450K DNA meth	–

The number of samples with matched RNA sequencing and 450K DNA methylation for each cancer type is given. The number of MethCORR genes for each cancer reflects the number of genes with *R*^2^ > 0.16 between observed and inferred RNA expression (iRNA) in both the discovery and the validation set, as previously defined for colorectal cancer [12]

Using MethCORR genes, a high intra-sample correlation was found between observed RNA expression and iRNA expression for the completely independent validation samples in all cancer types [Fig. 1b, c; median *R*^2^ all cancer types: 0.91 (*R*^2^ range all cancer types: 0.59–97; Additional file 3)]. Furthermore, for available independent validation samples with matched fresh-frozen and FFPE tissue samples (BRCA, PRAD, LUAD, BLCA, KIRC, and UCEC; 25 samples in total; Additional file 4), a significantly (*P* < 0.0001, Wilcoxon rank-sum test) higher intra-sample correlation between iRNA expression calculated from FFPE DNA methylation and matched fresh-frozen RNA sequencing profiles was found (median *R*^2^ all cancer types: 0.89; *R*^2^ range all cancer types: 0.69–0.95; Fig. 1d), compared to FFPE RNA sequencing and matched fresh-frozen RNA sequencing (median *R*^2^ all cancer types: 0.79; *R*^2^ range all cancer types: 0.49–0.88; Fig. 1d and Additional file 5: Fig. S1b). Demonstrating how inferring of RNA expression from FFPE DNA methylation often can be a superior route to obtain robust RNA expression profiles from FFPE tissue compared to direct sequencing of RNA extracted from FFPE tissue. The finding is in line with our previous observation in CRC [12]. A PCA provided additional evidence, by revealing that matched fresh-frozen and FFPE RNA sequencing profiles clustered according to preservation method, whereas samples clustered more according to cancer type when analyzing RNA sequencing of fresh-frozen samples and iRNA expression from matched FFPE samples (Fig. 1e).

The performance of the MethCORR method was compared to that of TOBMI [16] and BioMethyl [17], which are two alternative approaches to methylation-based RNA expression imputation. We compared the gene-specific model performance (inter-sample correlation between inferred RNA expression and observed RNA expression) for MethCORR, TOBMI, and BioMethyl and found that MethCORR exhibited overall higher inter-sample correlations (Additional file 5: Fig. S1c and S1d).

### MethCORR inferred RNA expression allows molecular stratification of breast, prostate, and lung cancer

Next, it was investigated whether iRNA expression could be used as a substitute for RNA expression in molecular stratification of breast, prostate, and lung cancer. These cancer types were in focus here due to the availability of 450K methylation profiles from independent fresh-frozen and FFPE samples, which allow comparative evaluation of the MethCORR method in both fresh-frozen and FFPE cohorts.

**Fig. 2:**
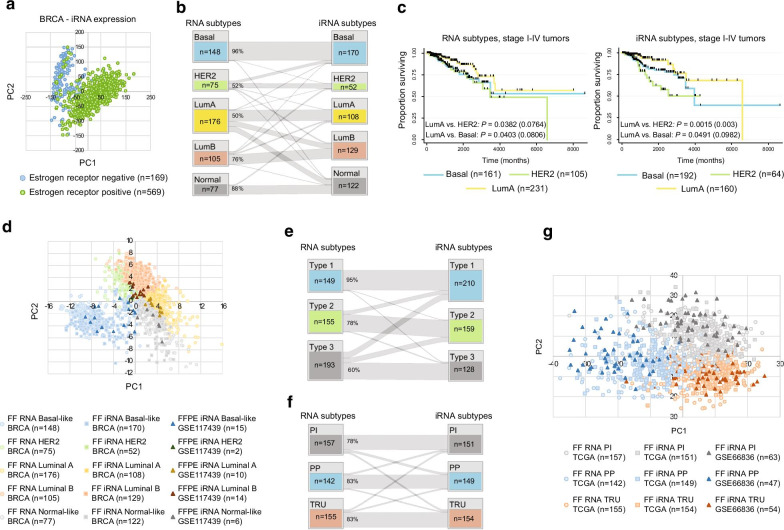
Molecular subtyping with MethCORR inferred RNA expression. **a** Scatterplot with the first- (PC1; X-axis) and second principal component (PC2; Y-axis) from a PCA performed with iRNA expression calculated for 738 TCGA BRCA samples with available estrogen receptor (ER) status. **b** Caleydo StratomeX [40] plot showing the concordance between PAM50 breast cancer subtype predictions made with RNA or iRNA expression as input for the TCGA BRCA cohort. Only samples with a confident subtype call for both input types are shown (*n* = 581; confidence = 1). **c** Kaplan–Meier plots showing the overall survival of AJCC stage I-IV patients from the TCGA BRCA cohort stratified according to PAM50 subtypes using either RNA-based (left panel) or iRNA-based (right panel) PAM50 subtype calls (for all confidence levels). Significance was evaluated by the log-rank test. In parenthesis is provided the Bonferroni-adjusted P values (two comparisons, i.e., LumA vs. HER2 and LumA vs. Basal). **d** Scatterplot with the first- (PC1; X-axis) and second principal component (PC2; Y-axis) from a PCA performed with TCGA BRCA RNA expression, TCGA BRCA iRNA expression, and iRNA expression calculated in an independent breast cancer FFPE cohort (GSE117439) for the PAM50 genes. Samples are colored according to their predicted subtype. Only samples with a confident subtype call are shown (confidence = 1). **e** Caleydo StratomeX plot showing the concordance between prostate cancer subtype predictions (ConsensusClusterPlus) made with RNA or iRNA expression as input for 497 TCGA PRAD tumor samples. **f** Caleydo StratomeX plot showing the concordance between lung cancer subtype predictions made with RNA or iRNA expression as input for 454 TCGA LUAD tumor samples. PI: Proximal Inflammatory, PP: Proximal Proliferative, TRU: Terminal Respiratory Unit. **g** Scatterplot with the first- (PC1; X-axis) and second principal component (PC2; Y-axis) from a PCA performed with TCGA LUAD RNA expression, TCGA LUAD iRNA expression, and iRNA expression calculated in an independent fresh-frozen cohort (GSE86836) for the 474 centroid genes used for subtyping. Samples are colored according to their predicted subtype

Initially, the focus was on breast cancer and a differential expression analysis with normal vs tumor tissue revealed an agreement of 95% in identified significantly differential expressed genes (*n* = 9936, Wilcoxon rank-sum test < 0.01) between RNA and iRNA expression (Additional file 6: Fig. S2a). This analysis underscores the ability of iRNA expression to identify possible biomarker candidates for discrimination and stratification of normal and tumor tissue samples. The clinical classification of breast cancer currently relies on histological grading, hormone receptor status, and molecular classification. For samples with available hormone receptor status on the estrogen receptor (ER), we found that samples clustered together according to receptor (ER) status both in a PCA and in bootstrap hierarchical clustering analysis performed with either RNA expression or iRNA expression in the TCGA BRCA cohort (Fig. 2a and Additional file 6:  Fig. S2b and S2c). This result supported and highlighted that iRNA expression in breast cancer possess biological information equally to RNA expression data. This separation of ER positive and negative samples was confirmed in a PCA and clustering analysis with iRNA expression calculated in an independent fresh-frozen (GSE84207) and, notably also, a FFPE cohort (GSE117439) with available ER status (Additional file 6: Fig. S2b and S2c).

In the clinical management of breast cancer, the PAM50 gene expression classifier is a widely used molecular classification system [18]. It stratifies the disease into the intrinsic subtypes “Basal-like,” “HER2-enriched,” “Luminal A,” “Luminal B,” and “Normal-like.” Here, all tumor samples of the TCGA BRCA cohort were PAM50 classified using either RNA expression or iRNA expression as input. A moderate concordance was observed between the subtype predictions made with RNA and iRNA expression data (Cohen’s kappa coefficient = 0.64; Fig. 2b). Concordance was observed for 72% of all analyzed samples and was low for the HER2-enriched and Luminal A subtype tumors (Basal-like 96%, HER2-enriched 52%, Luminal A 50%, Luminal B 76%, and Normal-like 88%; Fig. 2b). We also performed comparison of the RNA and iRNA-based PAM50 classification to the original microarray-based classification provided by the TCGA [3]. Here, a good agreement was found between microarray- and RNA-based classification, whereas the agreement between microarray and iRNA-based classification were again moderate (Additional file 6: Fig. S2d and S2e). To gain insights into possible reasons for PAM50 classification discrepancies, we performed an inspection of the 50 PAM50 genes. This showed that MethCORR model performance *R*^2^ was between 0.17 and 0.80 for these genes and that, e.g., top three genes defining the HER2-enriched subtype all had a *R*^2^ value below 0.37 (Additional file 6: Fig. S2f). We therefore speculate that disconcordance between RNA and iRNA PAM50 subtypes may be partly explained by MethCORR genes with low model performance. We next investigated if the PAM50 subtype classifications stratified patients into groups with differences in postoperative survival. Reports have shown that of the five subclasses, the Luminal A tumors have the most favorable prognosis, and the HER2-enriched and Basal tumors the worst [19, 20]. Indeed, despite differences in the RNA microarray-, RNAseq-, and iRNA-based PAM50 classifications, we found that all three classifications yielded overall survival estimates in agreement with this (Fig. 2c and Additional file 6: Fig. S2g). This may point to a relevance and prognostic potential of the iRNA-based PAM50 classification. While the clinical impact of the iRNA- and RNA-based classifications may be slightly different, we note that samples clustered by subtype status in a PCA regardless whether RNA or iRNA expression was used as input (Fig. 2d) and, moreover, also when the iRNA expression was inferred from fresh-frozen (TCGA BRCA cohort) and FFPE (GSE117439) samples (Fig. 2d). The latter highlights the robustness of MethCORR across tissue preservation methods.

To further illustrate the potential use of iRNA expression for molecular subtype discovery, we focused on prostate cancer. Molecular heterogeneity in prostate cancer has previously been addressed by molecular subtyping, in particular, the TCGA research network identified three molecular subtypes by unsupervised clustering of RNA expression profiles [21]. Here, we imitated the strategy used by the TCGA research network [21] and performed unsupervised clustering using both iRNA and RNA expression profiles. Indeed, three subtypes were identified, both when using TCGA PRAD iRNA expression or RNA expression data (Additional file 6: Fig. S2h). A good agreement between the iRNA and RNA derived subtypes was observed (Cohen’s kappa coefficient = 0.65; Fig. 2e; RNA Subtype 1 95%, RNA Subtype 2 78%, and RNA Subtype 3 60%). The findings were validated in independent FFPE samples (GSE73549). When the iRNA expression for the FFPE samples was analyzed together with the iRNA and RNA expression from the TCGA fresh-frozen samples, in a PCA, the samples generally clustered according to subtype status independently of preservation method (Additional file 6: Fig. S2i).

In lung cancer, Hayes et al. in 2006 proposed three adenocarcinoma transcriptional subtypes: Proximal Inflammatory (PI), Proximal Proliferative (PP), and Terminal Respiratory Unit (TRU) [22]. The subtypes, which had different molecular characteristics and differed in survival, have since been further characterized by the TCGA research network [6]. Applying the same subtyping strategy as the TCGA research network to RNA and iRNA expression values from the TCGA LUAD cohort revealed good concordance between the subtype predictions made with the different RNA inputs (Cohen’s kappa coefficient = 0.72; Fig. 2f; PI 78%, PP 83%, and TRU 83%). iRNA expression data were also extracted from the independent fresh-frozen lung cancer cohort (GSE66836) and used for subtyping. A combined PCA with iRNA expression data from GSE66836 together with iRNA and RNA expression data from TCGA LUAD revealed that the samples clustered according to subtype, independent of the study of origin and the input data type (Fig. 2g).

### MethCORR inferred RNA expression allows biological characterization of cancer subtypes and samples

**Fig. 3:**
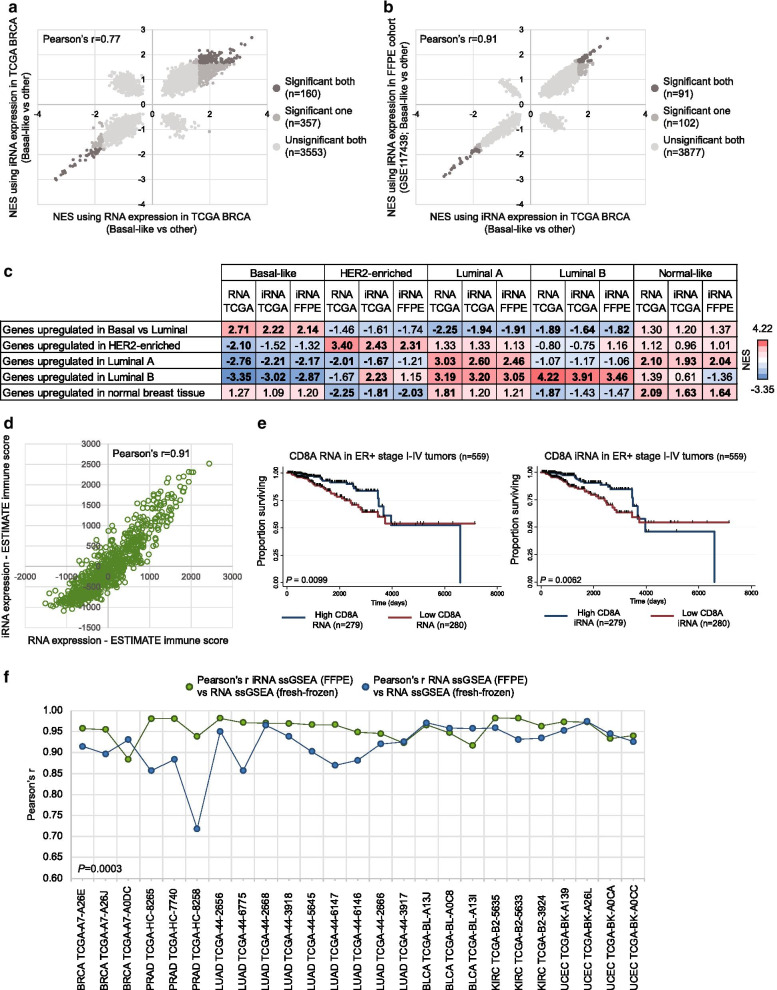
Subtype characterization with MethCORR inferred RNA expression. **a** Scatterplot with correlation between normalized enrichment scores (NESs) from a gene set enrichment analysis (GSEA) of the TCGA BRCA Basal-like subtype vs. all other BRCA samples (HER2-enriched, Luminal, and normal-like) performed with RNA expression (x-axis) or iRNA expression (y-axis). **b** Scatterplot with correlation between NESs from a GSEA of the TCGA BRCA Basal-like subtype vs. all other BRCA samples performed with iRNA expression as input (x-axis) and the Basal-like subtype vs. all other samples in the independent FFPE cohort (GSE117439) performed with iRNA expression as input (y-axis). **c** Table with selected gene sets differentially enriched between breast cancer subtypes identified by GSEA analysis. GSEA was performed with RNA and iRNA expression in the TCGA BRCA cohort and iRNA expression in the independent breast cancer FFPE cohort (GSE117439). Gene sets with a positive NES are indicated by a red color scale and gene sets with a negative NES are indicated by a blue color scale. Significance is highlighted by bold (FDR < 0.05). See the methods section for the origin of the selected gene sets. **d** Scatterplot with correlation between ESTIMATE immune scores from an ESTIMATE [46] analysis performed with RNA expression (x-axis) or iRNA expression (y-axis). **e** Kaplan–Meier plots showing the overall survival of AJCC stage I-IV ER positive patients from the TCGA BRCA cohort stratified according to high or low CD8A expression (median cut-off) using either RNA (left panel) or iRNA (right panel). Significance was evaluated by the log-rank test. **f** Line chart with correlations between enrichment scores from single sample GSEA performed with RNA expression in fresh-frozen tissue and iRNA expression calculated in matched FFPE tissue or RNA expression in matched FFPE tissue. Correlations are shown for all samples with matched tissue for the BRCA (*n* = 3), PRAD (*n* = 3), LUAD (*n* = 9), BLCA (*n* = 3), KIRC (*n* = 3), and UCEC (*n* = 4) cohorts

Although categorical subtyping is a potential clinical relevant strategy for molecular stratification of cancer, it does not capture all aspects of inter-tumor heterogeneity. Therefore, to illustrate that iRNA expression allows more extensive and uniform biological characterization of fresh-frozen and FFPE samples a comparative molecular characterization of the breast cancer subtypes was performed by gene set enrichment analysis (GSEA). Initially, it was investigated if GSEA identified the same gene set enrichments when performed with iRNA as input as with RNA as input. Indeed, a high correlation in the normalized enrichment scores (NESs) was observed for most gene sets in all five breast cancer PAM50 subtypes (Fig. 3a and Additional file 7: Fig. S3a; Pearson’s r range all subtypes: 0.65–0.91). Furthermore, a high concordance for most gene sets was also observed when comparing NESs from GSEA of BRCA subtypes and subtypes predicted in the independent FFPE cohort (GSE117439) performed with iRNA expression as input (Fig. 3b and Additional file 7: Fig. S3b; Pearson’s r range all subtypes: 0.59–0.96). A focused analysis of the five key gene sets known to be enriched in each subtype showed that “Genes upregulated in Basal-like vs Luminal” were significantly enriched in Basal-like, “Genes upregulated in HER2-enriched” were significantly enriched in HER2-enriched, “Genes upregulated in Luminal A/B” were significantly enriched in Luminal A/B, and “Genes upregulated in normal breast tissue” were significantly enriched in Normal-like (Fig. 3c). These findings were similar independent of the study of origin and the input data type (Fig. 3c). Additionally, when focusing on characterization of the tumor immune microenvironment, a high correlation was found between tumor immune infiltration abundance scores determined with RNA and iRNA expression (Fig. 3d). Notably, the prognostic T-lymphocyte marker CD8A was able to stratify the major group of estrogen receptor positive (ER+) breast cancer patients into groups with high and low survival risk independently of using RNA or iRNA expression and a similar trend was seen when analyzing all breast cancer samples (Fig. 3e and Additional file 7: Fig. S3c).

Finally, single sample GSEA (ssGSEA) was performed for the 25 samples (from six cancer types) with matched fresh-frozen RNA expression data and FFPE RNA expression/iRNA expression data. Overall, high correlations between matched fresh-frozen and FFPE ssGSEA enrichment scores were observed, regardless of comparing fresh-frozen RNA expression with FFPE RNA expression or FFPE iRNA expression (Fig. 3f). Notably, we found that correlations between enrichment scores for ssGSEA performed with matched fresh-frozen RNA expression and FFPE iRNA expression were significantly higher (average Pearson’s *r* = 0.96; *P* = 0.0003, Wilcoxon rank-sum test) as compared to ssGSEA performed with FFPE RNA expression data (average Pearson’s *r* = 0.92; Fig. 3f).

## Discussion

In this study, we investigated if the MethCORR method, originally developed for CRC, would be applicable in other cancer types to infer gene expression information from DNA methylation, as an alternative to direct RNA profiling. We applied the MethCORR method to ten common cancer types and were able to infer RNA expression with good accuracy for both fresh-frozen and FFPE tissue samples. The conversion of DNA methylation profiles into a gene-centric expression format represent a particular strength for molecular analysis as it allows analysis of fresh-frozen and FFPE samples using the plethora of bioinformatics tools, databases, signatures, and biomarkers that is established for RNA expression data. We illustrate this by showing that iRNA expression can be used as a substitute for RNA expression during molecular classification/subtyping of three cancer types (Fig. 2) and during pathway analysis by GSEA independently of the sample preservation type (Fig. 3). In contrast, such molecular analysis is currently not possible using DNA methylation data.

Transcriptional profiling can be difficult in FFPE tissue due to poor quality of the extracted RNA, especially of archival samples [8]. Using MethCORR, we have previously shown for colorectal cancer that fresh-frozen samples with low-quality RNA exhibit poorer intra-sample iRNA-RNA correlations than samples with high RNA quality [12]. This analysis shows that even slight RNA degradation in fresh-frozen tissue can lead to poor correlations between iRNA and RNA, an effect that is expected to be much more pronounced in FFPE and fresh-frozen sample comparisons. In support of this, we here confirm for TCGA samples from six cancers that correlation between transcriptome profiles from matched fresh-frozen and FFPE samples was modest (Fig. 1d). This can preclude confident transcriptional profiling and molecular analysis of archival FFPE samples with long-term clinical follow-up information and of FFPE samples that are routinely collected for all cancer patients in clinical departments. We and others have reported that DNA methylation is robustly measured in FFPE tissue [12–15], which is likely due to the fact that DNA is considered to have a higher biostability compared to RNA in FFPE tissue [23, 24] and that DNA methylation is robustly measured in FFPE tissue by the Illumina Infinium BeadChip platform (450K/EPIC) upon DNA restoration [13, 14]. Consistently, the present matched analyses of FFPE and fresh-frozen tissues from six cancer types showed that MethCORR inferred RNA expression in FFPE tissue was most frequently better correlated to RNA sequencing data from fresh-frozen tissue, than RNA sequencing data of the FFPE tissue (Fig. 1). This demonstrates how MethCORR analysis may unlock the great clinical potential of archival as well as routinely collected FFPE samples.

We fully acknowledge, and are encouraged, that some studies have shown good performance of RNA expression profiling in FFPE tissue and that such data have been used for transcriptional analysis [25–28]. However, only relatively few samples have been included in these studies and we are not aware of any large RNA sequencing studies performed on FFPE cohorts, which may indicate that FFPE RNA sequencing procedures still need improvements to become widely used. Optimization of the FFPE preservation conditions and newer sequencing technologies have been introduced since the TCGA project was undertaken, which may improve FFPE RNA sequencing and outperform MethCORR. However, large datasets with newer RNA sequencing data and matched DNA methylation data are currently not available to allow such comparisons. We note that the performance of FFPE RNA sequencing [11] and transcription-based subtyping [27] is strongly dependent on the time passed since formalin fixation. It appears that the negative effects of fixation increase as time passes by. Therefore, it remains to be established if the pronounced shift in RNA expression profiles from fresh-frozen to FFPE tissue [29] can be fully resolved.

The analyses presented here show that the FFPE has less effect on DNA methylation and inference of RNA expression. The matched FFPE iRNA and fresh-frozen RNA sequencing profiles clustered together according to cancer type rather than preservation type, whereas matched fresh-frozen and FFPE RNA sequencing profiles clustered according to preservation type rather than phenotypic differences (Fig. 1e) [12, 30, 31]. This indicates that the biological information in DNA methylation/iRNA expression is less influenced by the preservation method.

Collectively, our detailed analysis of breast, lung, and prostate cancer shows that MethCORR iRNA expression profiles can support both uniform subtype classification, unsupervised subtype discovery, and characterization in fresh-frozen and FFPE samples using bioinformatics tools normally employed for RNA expression analysis. We find an overall 76% correspondence in molecular subtype calls for breast, prostate, and lung cancer when using RNA and iRNA expression profiles as input. We acknowledge that the concordance for some subtypes (especially the BRCA HER2-enriched and Luminal A subtypes) may be considered modest. We speculate that differences in MethCORR modeling accuracy between genes may be a contributor to differences in subtype classification when comparing RNA sequencing, RNA microarray, and iRNA data. This is not entirely unexpected nor disqualifying, as methods for subtype classification have previously been modified to better fit the profiling approach. For example, the original consensus molecular classifier for colorectal tumors based on RNA microarray data [2] was recently adapted to RNA sequencing data from, e.g., cell lines by replacement of genes included in the classifier [32]. Still, it is encouraging that iRNA-based PAM50 classification recapitulates well-established survival differences between breast cancer subtypes despite disconcordances between RNA and iRNA-based subtype calls. However, further studies are needed to further evaluate the potential of iRNA-based PAM50 subtyping, as illustrated here, in independent cohorts.

We compared the performance of MethCORR to the TOBMI [16] and BioMethyl [17] methods and found that MethCORR outperformed both of the methods. The BioMethyl method bears high resemblance to MethCORR; however, BioMethyl only uses CpG sites associated with each gene (i.e., CpG sites in the gene region) to infer gene expression, whereas MethCORR identified correlations genome-widely. Perhaps for this reason, we found that the MethCORR method has overall better performance in RNA expression imputation. Also, the MethCORR models are only trained once and can hereafter be directly applied to independent DNA methylation datasets to infer gene expression. In contrast, TOBMI [16] is a k-nearest neighbor weighted method, and therefore, re-computation should be performed whenever inferring of gene expression is performed for new samples.

Finally, it should be noticed that a major limitation is the requirement of large cohorts with matched DNA methylation and RNA sequencing data for establishment of MethCORR models within a specific cancer type. However, with this manuscript we provide pre-established MethCORR models for ten common cancer types, which will allow direct use of MethCORR for these cancers. Furthermore, it should be noted that MethCORR allows three layers of molecular information to be derived from a single DNA methylation profile: the methylome profile itself (generated by either of the 450K or EPIC methylation arrays [12]), a MethCORR inferred RNA expression profile, and a chromosome copy number profile, calculated from the methylation array signal intensity [33]. Consequently, MethCORR is a cost-efficient alternative method to RNA sequencing with robust performance in FFPE tissue.

## Conclusions

We have demonstrated that MethCORR can infer RNA expression from DNA methylation profiles in all ten cancer types analyzed, in addition to CRC where the method was originally developed [12]. Furthermore, we have shown that inferred RNA expression allows subtype discovery, classification, and characterization of fresh-frozen and FFPE samples. Hereby, we envision that MethCORR inferred gene expression profiles can contribute to testing of molecular classification and biomarkers both in a clinical setting using the FFPE tissue that is standardly collected from all cancer patients, and in unexplored FFPE archives. With this study, the use of MethCORR by the scientific community is facilitated, as the ten MethCORR matrixes and the associated gene regression models are made freely available, which allow calculation of inferred RNA expression profiles in independent samples with available DNA methylation data.

## Methods

### Cancer cohorts and datasets

The BRCA, PRAD, LUAD, LUSC, STAD, SKCM, BLCA, KIRC, ESCA, and UCEC cohorts were all collected as part of the TCGA Project. All cohorts consist of mucosa and tumor samples. All clinical information, RNA sequencing data, and DNA methylation data were acquired via the UCSC XENA public Data Hubs [34] (https://xena.ucsc.edu/public/) and the GDC data portal [35] (https://portal.gdc.cancer.gov/). The GSE117439, GSE84207, GSE66836, and GSE73549 cohorts were acquired as series matrix files from the Gene Expression Omnibus (GEO) [36]. Only tumor samples were used for subtype, biomarker, and pathway analysis (Figs. 2 and 3). The estrogen receptor status for BRCA samples was obtained from the column “breast_carcinoma_estrogen_receptor_status” in the “Phenotypes” file (clinical matrix) available from the UCSC XENA database [34].

### DNA methylation data

Infinium HumanMethylation450K BeadChip (HM-450K) DNA methylation profiles for TCGA cohort samples were acquired from the UCSC XENA Public Data Hubs [34] (https://xena.ucsc.edu/public/) and GDC data portal [35] (https://portal.gdc.cancer.gov/). HM-450K DNA methylation profiles for the GSE117439, GSE84207, GSE66836, and GSE73549 cohorts were acquired as series matrix files from the Gene Expression Omnibus (GEO) [36]. Methylation profiles were acquired as normalized DNA methylation β-values.

### RNA sequencing data

RNA sequencing profiles for TCGA cohort samples were acquired from the UCSC XENA Public Data Hubs [34] (https://xena.ucsc.edu/public/) as log2(FPKM + 1) normalized RNA expression values for 20,530 genes and via the GDC data portal [35] (https://portal.gdc.cancer.gov/) as FPKM normalized RNA expression values for 60,483 genes.

### Datasets used for the MethCORR method

The MethCORR method [12] was independently applied to ten TCGA cancer types, which established a MethCORR matrix and linear regression models specific to each cancer type. Primarily, MethCORR development was performed using HM-450K DNA methylation data and RNA sequencing data acquired in normalized format via the UCSC XENA Public Data Hubs (https://xena.ucsc.edu/public/) [34].

Secondarily, MethCORR development was performed using HM-450K DNA methylation data and RNA sequencing data (17,611 RNAs, these were selected from the original dataset of 60,483 genes as they overlap with the RNAs included in the UCSC XENA RNA dataset) acquired in normalized format from the GDC data portal [35] (https://portal.gdc.cancer.gov/). This analysis was performed to generate a GDC data-based MethCORR matrix that was used for analysis of the matched TCGA fresh-frozen and FFPE samples included in this study (BRCA, PRAD, LUAD, BLCA, KIRC, and UCEC), as data from these samples were acquired via the GDC data portal (Additional file 4). All samples from the GDC database with matched fresh-frozen and FFPE samples were excluded from training of MethCORR and only used for independent validation. During correlation analysis with RNA-sequencing data from matched fresh-frozen and FFPE samples, only data that originated from the same source center (RNA sequencing center; information available at https://portal.gdc.cancer.gov/) and subjected to identical bioinformatics processing were analyzed. One fresh-frozen-FFPE dataset pair was excluded as fresh-frozen and FFPE RNA sequencing datasets did not originate from the same source center. Furthermore, in the cases where RNA sequencing was performed on two fresh-frozen samples for a patient, the RNA seq. run where the fresh-frozen sample was analyzed on the same sample plate as the matched FFPE sample was selected (information from the TCGA barcode).

### The MethCORR method—identification of RNA expression-correlated CpG sites

Identification of expression-correlated CpG sites was performed as previously described [12]: each cancer type was divided in two discovery sets (set 1–2, each encompassing 40% of samples), whereas a third set was reserved for independent validation (set 3, 20% of the samples; Additional file 1). Genome-wide Spearman correlations between the expression of each RNA and the DNA methylation level (β-value) of each CpG site were calculated independently in each discovery set. All nonsignificant Spearman correlation pairs were discarded. The remaining significant (*P* < 0.01) expression-correlated CpG sites were ranked by their Spearman’s rho in each discovery set and after that by their rank-sum within the discovery set 1 and 2 to identify “common” top expression-correlated CpG sites. From these common ranked CpG site lists, we selected up to 100 CpG sites whose methylation level (β-value) most negatively or positively correlated with RNA expression, which resulted in lists of ≤ 200 expression-correlated CpG sites specific for each RNA (depending on the number of expression-correlated CpG sites in the common ranked lists). The ≤ 200 expression-correlated CpG sites specific for each RNA constitutes the MethCORR matrix (UCSC XENA-based MethCORR matrix specific for each cancer is integrated in the provided MethCORR v1.0 R workspace (https://moma.dk/MethCORR-software); GDC-based MethCORR matrix available upon request).

### The MethCORR method—calculation of MethCORR scores

For each sample in each cancer type, we used the methylation β-values of the gene-specific top ≤ 200 expression-correlated CpG sites, included in the cancer specific MethCORR matrix, to calculate a MethCORR score (MCS) for each gene using the formula [12]:

$${\text{MCS}} = \frac{1}{ \leq 200}\left( {\mathop \sum \limits_1^{ \leq 100} \beta \,{\text{value}}\,{\text{pos.}}\,{\text{correl.}}\,{\text{CpG}}\,{\text{probe}} + \mathop \sum \limits_1^{ \leq 100} 1 - \beta \;{\text{value}}\,{\text{neg.}}\,{\text{correl.}}\,{\text{CpG}}\,{\text{probe}}} \right).$$

The MCS formula calculates the average methylation value of the expression-correlated CpG sites specific for each gene. The R-package Impute v1.62.0 [39] was used to impute MCSs in samples with missing MCSs.

### The MethCORR method—modeling of RNA expression and calculation of inferred RNA expression

For each cancer, we modeled the relationship between MCSs and RNA expression for each gene in the discovery set samples (set 1 + 2). We used both simple linear (RNA = *B*_0_ + *B*_1_*MCS) and polynomial regression models (RNA = *B*_0_ + *B*_1_*MCS + *B*_2_*MCS^2^… + *B*_n_*MCS^*n*^; *n* = 2–4). The Caret R-package v6.0-86 [37] was used to perform modeling by 10× 10-fold cross-validation. The best model was selected based on the root mean square error (RMSE). The model performances were highly similar for simple linear and polynomial regression models for most genes and, therefore, polynomial regression models were only selected if a ≥ 5% relative decrease in RMSE was observed over simple linear regression models. The gene-specific model performances were independently validated in validation set 3 (UCSC XENA model performance in Additional file 2). Genes with well-performing models (*R*^2^ > 0.16) in the discovery sets and the validation set were regarded as MethCORR genes and were included in the MethCORR matrix. All poorer performing models (*R*^2^ ≤ 0.16) were excluded. These criteria for MethCORR gene annotation are in line with our previous MethCORR analysis of colorectal cancer [12]. For each gene in each sample, we used MCSs as input in the gene-specific regression model to infer RNA expression (iRNA expression).

The UCSC XENA linear regression models are integrated in the provided MethCORR v1.0 R workspace (https://moma.dk/MethCORR-software). The MethCORR R workspace provides instructions on how to predict iRNA expression from user-provided Illumina Human methylation 450K or EPIC datasets of either of the 11 cancer types we have analyzed by MethCORR. Example 450K data from the UCSC XENA database is provided for each cancer type. The successful application of MethCORR on Human Methylation EPIC data have been described previously for colorectal cancer samples [12]. The MethCORR v1.0 R workspace was developed using R version 4.0.0, the “data.table” R package version 1.12.8 [38] and the “impute” R package version 1.62.0 [39]. GDC linear regression models are available upon request.

### TOBMI

The R package TOBMI [16] was used to infer RNA expression in validation set 3 samples for all ten cancers. We used HM-450K DNA methylation and RNA sequencing profiles from the UCSC XENA Public Data Hubs [34] (https://xena.ucsc.edu/public/) as input and default method parameters. The gene-specific model performance (inter-sample correlation between inferred RNA expression and observed RNA expression) was evaluated for overlapping genes between TOBMI and MethCORR.

### BioMethyl

The R package BioMethyl v1.1 [17] was used to infer RNA expression in all overlapping samples between BioMethyl and MethCORR for all ten cancers. We used HM-450K DNA methylation profiles from the UCSC XENA Public Data Hubs [34] (https://xena.ucsc.edu/public/) as input and default method parameters. We used RNA expression profiles from Firehose (https://gdac.broadinstitute.org/) to evaluate the gene-specific model performance (inter-sample correlation between inferred RNA expression and observed RNA expression) for genes overlapping between BioMethyl and MethCORR. The RNA expression profiles were log2(RSEM + 1) transformed followed by a z-transformation across samples as described by Wang et al. [17].

### AUC analysis

AUC analysis was performed using the R-package ROCR v1.0–11 with RNA expression or iRNA expression data as input.

### Principal component analysis

Principal component analysis (PCA) was performed using the R-package Stats v3.6.0 with RNA expression or iRNA expression data as input.

### Bootstrap hierarchical clustering

Bootstrap clustering was performed to evaluate the stability of ER positive and ER negative breast cancer tumor clusters using the R package pvclust v2.2-0, 1000 repetitions and Ward.D2 linkage. Clustering was performed with RNA or iRNA expression as input, and a row standard score was calculated with the scale function for each gene. AU (Approximately Unbiased) values were analyzed to evaluate clustering stability and clusters with AU > 0.9 are considered highly stable.

### Caleydo StratomeX

Caleydo StratomeX [40] analysis was performed to visualize concordance between subtype predictions using the Caleydo v3.1.5 software.

### PAM50 classification

Subtype classification of breast cancer cohorts was performed using the 50-gene PAM50 predictor [18]. Classifications were performed with RNA expression or iRNA expression as input. Microarray-based PAM50 annotations were taken directly from the “Phenotypes” file from the TCGA BRCA project available at the UCSC XENA database (https://xena.ucsc.edu/public/).

### ConsensusClusterPlus classification

Subtype classification of prostate cancer cohorts was performed by Consensus average linkage hierarchical clustering using the R package ConsensusClusterPlus v1.48.0 [41]. The top 3000 most variable genes were selected by median absolute deviation for both RNA expression data and iRNA expression data. Input data were gene median centered.

### Nearest centroid predictor classification

Subtype classification of lung cancer cohorts was performed using previously published gene expression subtype predictor centroids [42]. RNA expression and iRNA expression data were gene median centered for genes common to the predictor (474 out of 509 predictor genes). The maximum Pearson’s correlation coefficient between class predictor centroids and sample RNA expression or iRNA expression was used for subtype assignment.

### Gene set enrichment analysis

Gene set enrichment analysis (GSEA) was performed with RNA expression and iRNA expression using the GSEA 4.1 tool [43] with default settings and gene-set permutation type. The Molecular Signatures Database (MsigDB) gene set C2 collection 7.2 was used. The following gene sets were used for biological characterization: “Genes upregulated in Basal-like vs Luminal (CHARAFE_ BREAST_CANCER_LUMINAL_VS_BASAL_DN),” “Genes upregulated in HER2-enriched (SMID_BREAST_CANCER_ERBB2_UP),” “Genes upregulated in Luminal A (SMID_BREAST_CANCER_LUMINAL_A_UP),” “Genes upregulated in Luminal B (SMID_BREAST_CANCER_LUMINAL_B_UP),” and “Genes upregulated in normal breast tissue (TURASHVILI_BREAST_LOBULAR_CARCINOMA_VS_DUCTAL_NORMAL_DN).”

Single sample GSEA [44] was performed with RNA expression and iRNA expression data as input using the ssGSEAProjection v9.1.2 GenePattern module [45] and the Molecular Signatures Database (MsigDB) gene set C2 collection 7.0.

### Estimate

ESTIMATE Immune scores were calculated using the R-package ESTIMATE v1.0.13 [46] using default parameters, RNA, and iRNA expression as input.

### Statistical analysis

Statistical significance of differences between groups was determined using a nonparametric Wilcoxon rank-sum test. During GSEA, a false discovery rate (FDR) < 0.05 was considered significant. Overall survival (OS) analysis was performed using the Kaplan–Meier method with the Stata/IC 14.2 (StataCorp) software. Significance was evaluated by log-rank test of equality in TNM stage I-IV TCGA BRCA tumors using curated clinical follow-up information [47]. Log-rank P values were adjusted using the Bonferroni correction method when multiple comparisons were made in survival analysis. Samples with no clear TNM stage annotation, incomplete OS survival information, and redacted samples were excluded from the survival analysis.

## Supplementary information

**Additional file 1: Table S1.** List of UCSC XENA TCGA samples used for the development of the MethCORR matrix and regression models.

**Additional file 2: Table S2.** Gene-specific model fit for UCSC XENA TCGA BRCA. **Table S3.** Gene-specific model fit for UCSC XENA TCGA PRAD. **Table S4.** Gene-specific model fit for UCSC XENA TCGA LUAD. **Table S5.** Gene-specific model fit for UCSC XENA TCGA LUSC. **Table S6.** Gene-specific model fit for UCSC XENA TCGA STAD. **Table S7.** Gene-specific model fit for UCSC XENA TCGA BLCA. **Table S8.** Gene-specific model fit for UCSC XENA TCGA SKCM. **Table S9.** Gene-specific model fit for UCSC XENA TCGA KIRC. **Table S10.** Gene-specific model fit for UCSC XENA TCGA UCEC. **Table S11.** Gene-specific model fit for UCSC XENA TCGA ESCA.

**Additional file 3: Table S12.** UCSC XENA TCGA intra-sample (per sample) specific model fit. Data is shown per individual sample and as summarized results.

**Additional file 4: Table S13.** GDC database UUIDs and TCGA barcodes for data files (https://portal.gdc.cancer.gov/) from 25 patients with available RNA-seq. and 450K DNA methylation profiles from matched fresh-frozen and FFPE tissues.

**Additional file 5: Figure S1.** MethCORR inferred RNA expression in ten cancer types. **a)** Graph showing the inter-sample RNA expression-iRNA expression squared correlations (R^2^) for BRCA, PRAD, and LUAD validation samples. Genes are ranked according to increasing RNA expression standard deviation in discovery samples. **b)** Scatterplots with correlations between RNA expression in matched fresh-frozen tissue and FFPE tissue for a representative validation sample from the TCGA BRCA, PRAD, and LUAD cohorts. **c)** Boxplot with MethCORR and TOBMI [16] validation set 3 inter-sample RNA expression-iRNA expression squared correlations (R^2^) for overlapping genes between the two methods. Data for all ten cancers are shown. * Wilcoxon rank-sum p<10^−10^. **d)** Boxplot with MethCORR validation set 3 and BioMethyl [17] inter-sample RNA expression-iRNA expression squared correlations (R^2^) for overlapping genes between the two methods. Data for all ten cancers are shown. * Wilcoxon rank-sum p<10^−5^.

**Additional file 6: Figure S2.** Molecular subtyping with MethCORR inferred RNA expression. **a)** Scatterplot with correlation between AUC values from a tumor vs normal analysis performed with RNA expression (x-axis) or iRNA expression (y-axis). **b)** Scatterplot with the first principal component (PC1; X-axis) and the second principal component (PC2; Y-axis) from a PCA performed with (left) RNA expression from TCGA BRCA samples, (middle) iRNA expression calculated in an independent fresh-frozen (GSE84207) cohort, and (right) iRNA expression calculated in an independent FFPE (GSE117439) cohort. Samples are colored according to their estrogen receptor (ER) status. **c)** Cluster dendrograms from hierarchical boostrap clustering (1000 repetitions) performed with BRCA RNA expression, BRCA iRNA expression, and iRNA expression from the fresh-frozen GSE84207 cohort, and the FFPE GSE117439 cohort. Samples with a “long id name” are ER negative samples. Approximately unbiased p-values (AU) values are given for each cluster node and clusters with AU>0.9 are highlighted by pink rectangles. **d+e)** Caleydo StratomeX [40] plots showing the concordance between TCGA BRCA microarray based PAM50 subtypes and RNA **(d)** or iRNA **(e)** expression based PAM50 subtypes (confidence=1). **f)** Scatterplot with regression model performance R^2^ (in independent validation samples) for the 50 genes that constitutes the PAM50 subtype classifier. Top three genes with the highest centroid value is marked for each PAM50 subtype. **g)** Kaplan–Meier plot showing the overall survival of AJCC stage I-IV patients from the TCGA BRCA cohort stratified according to microarray-based PAM50 subtypes (left panel), RNA-based PAM50 subtypes with confidence call=1 (middle panel), and iRNA-based PAM subtypes with confidence call=1 (right panel). Significance was evaluated by the log-rank test. In parenthesis is provided the Bonferroni-adjusted P values (two comparisons, i.e., LumA vs. HER2 and LumA vs. Basal). **h)** Consensus cumulative distribution function (CDF) plots for ConsensusClusterPlus analysis performed with (left) iRNA expression and (right) RNA expression for 497 TCGA PRAD tumor samples. The number of clusters, k, is determined where the CDF first approaches maximum [41, 48]. Here, a large increase is seen between k=2 and k=3 and further increases in k does not improve consensus substantially, i.e., k=3 for both iRNA expression and RNA expression. **i)** Scatterplot with the first principal component (PC1; X-axis) and the third principal component (PC3; Y-axis) from a PCA performed with TCGA PRAD RNA expression, TCGA PRAD iRNA expression, and iRNA expression calculated in an independent prostate cancer FFPE cohort (GSE73549). Samples are colored according to their predicted subtype.

**Additional file 7. Figure S3.** Subtype characterization with MethCORR inferred RNA expression. **a)** Scatterplots showing correlations between normalized enrichment sores (NESs) from a gene set enrichment analysis (GSEA) of the TCGA BRCA HER2-enriched subtype vs. all other BRCA samples, the TCGA BRCA Luminal A subtype vs. all other BRCA samples, the TCGA BRCA Luminal B subtype vs. all other BRCA samples, and the TCGA BRCA Normal-like subtype vs all other BRCA samples performed with RNA expression (x-axis) and iRNA expression (y-axis). **b)** Scatterplots showing correlations between NESs from a GSEA of the HER2-enriched subtype vs. all other samples, the Luminal A subtype vs. all other samples, the Luminal B subtype vs. all other samples, and the Normal-like subtype vs all other samples performed with iRNA expression in the TCGA BRCA cohort (x-axis) and iRNA expression in the independent breast cancer FFPE cohort (GSE117439; y-axis). **c)** Kaplan–Meier plots showing the overall survival of all AJCC stage I-IV patients from the TCGA BRCA cohort stratified according to high or low CD8A expression (median cut-off) using either RNA (left panel) or iRNA (right panel). Significance was evaluated by the log-rank test.

## Data Availability

The RNA sequencing data and HM-450K DNA methylation datasets from the TCGA cancer cohorts analyzed during the current study are available from the UCSC XENA Public Data Hubs [34] (https://xena.ucsc.edu/public/) and the GDC data portal [35] (https://portal.gdc.cancer.gov/). The GSE117439, GSE84207, GSE66836, and GSE73549 HM-450K DNA methylation datasets analyzed during the current study are available from the Gene Expression Omnibus database repository [36]. All data generated during this study are included in the published article and its supplementary information files.

## Abbreviations

CRC: Colorectal cancer
ER: Estrogen receptor
FDR: False discovery rate
FFPE: Formalin-fixed, paraffin-embedded
GSEA: Gene set enrichment analysis
HM-450K: Infinium HumanMethylation450K BeadChip
iRNA: Inferred RNA expression
MCS: MethCORR score
NES: Normalized enrichment score
OS: Overall survival
PCA: Principal component analysis
RMSE: Root mean square error
ssGSEA: Single sample GSEA
TCGA: The Cancer Genome Atlas
