# Supplementary figures and images for: MethCORR infers gene expression from DNA methylation and allows molecular analysis of ten common cancer types using fresh-frozen and formalin-fixed paraffin-embedded tumor samples
Source: Clin Epigenetics. 2021 Jan 28;13:20. doi: 10.1186/s13148-021-01000-0 (PMC7842045; doi:10.1186/s13148-021-01000-0)

Supplementary Figure 1

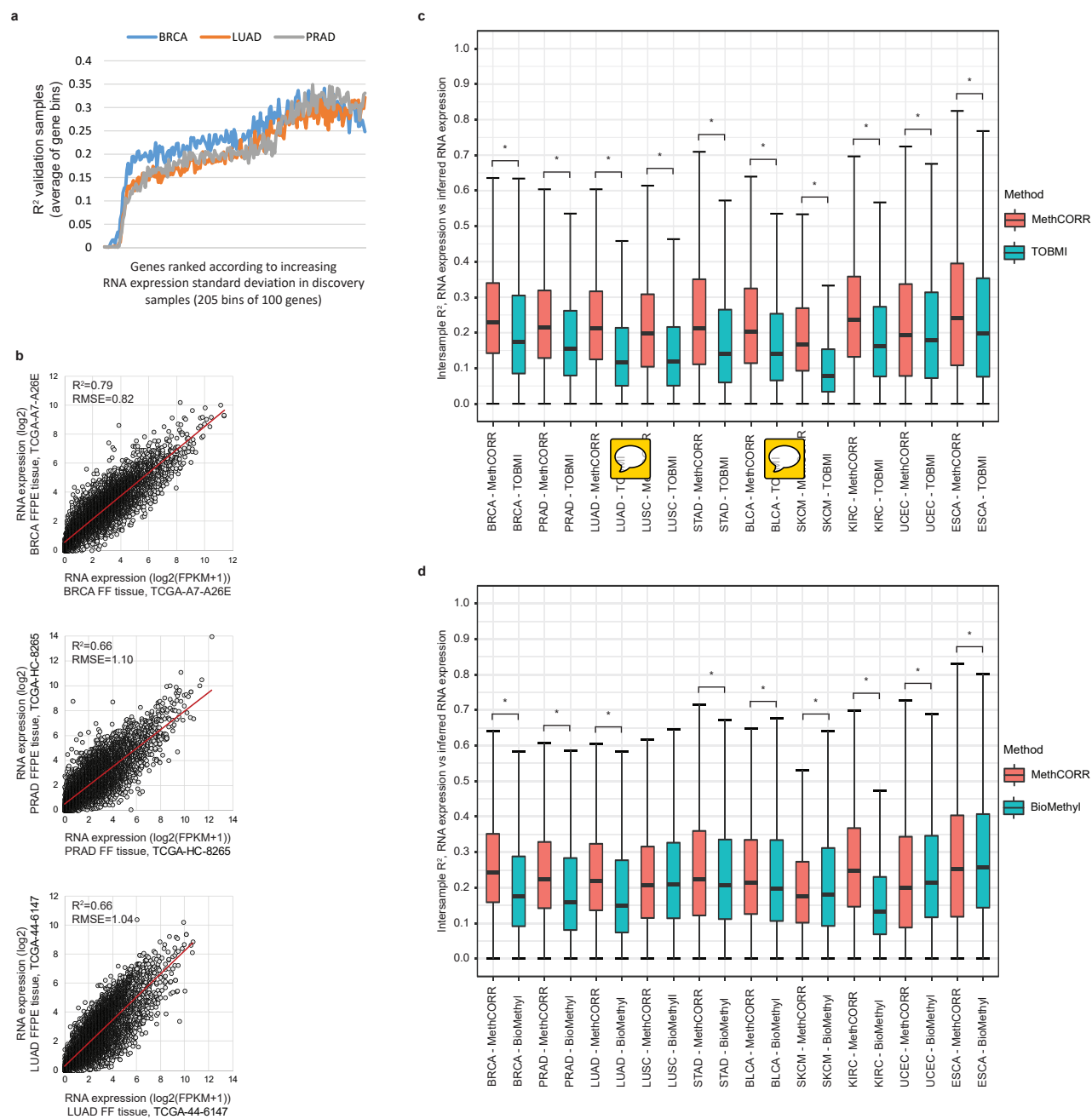

Supplement: Supplementary file 5 — Additional file 5: Figure S1. MethCORR inferred RNA expression in ten cancer types. a) Graph showing the inter-sample RNA expression-iRNA expression squared correlations (R2) for BRCA, PRAD, and LUAD validation samples. Genes are ranked according to increasing RNA expression standard deviation in discovery samples. b) Scatterplots with correlations between RNA expression in matched fresh-frozen tissue and FFPE tissue for a representative validation sample from the TCGA BRCA, PRAD, and LUAD cohorts. c) Boxplot with MethCORR and TOBMI [16] validation set 3 inter-sample RNA expression-iRNA expression squared correlations (R2) for overlapping genes between the two methods. Data for all ten cancers are shown. * Wilcoxon rank-sum p<10−10. d) Boxplot with MethCORR validation set 3 and BioMethyl [17] inter-sample RNA expression-iRNA expression squared correlations (R2) for overlapping genes between the two methods. Data for all ten cancers are shown. * Wilcoxon rank-sum p<10−5. [file 13148_2021_1000_MOESM5_ESM.pdf]

Supplementary Figure 2

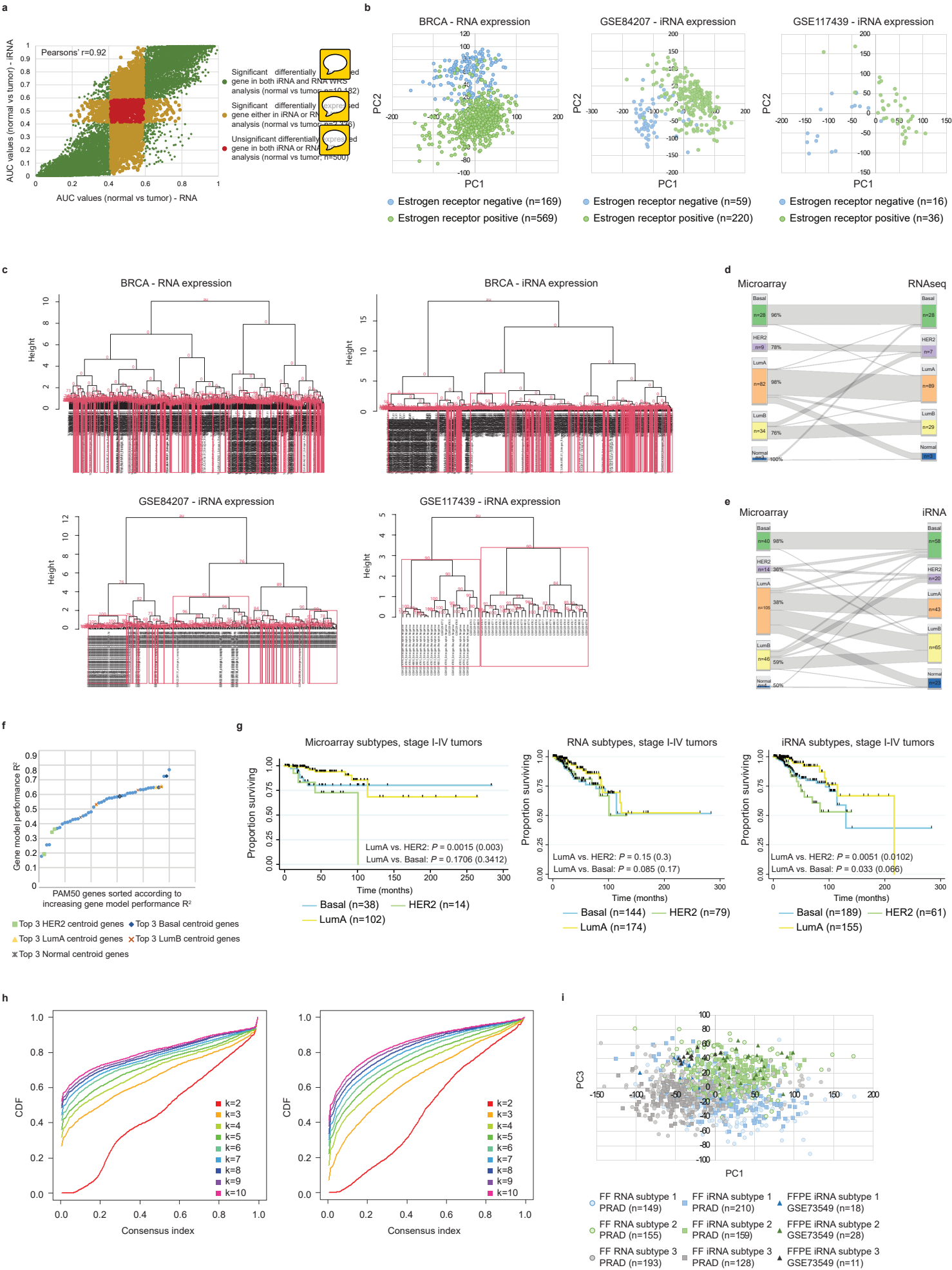

Supplement: Supplementary file 6 — Additional file 6: Figure S2. Molecular subtyping with MethCORR inferred RNA expression. a) Scatterplot with correlation between AUC values from a tumor vs normal analysis performed with RNA expression (x-axis) or iRNA expression (y-axis). b) Scatterplot with the first principal component (PC1; X-axis) and the second principal component (PC2; Y-axis) from a PCA performed with (left) RNA expression from TCGA BRCA samples, (middle) iRNA expression calculated in an independent fresh-frozen (GSE84207) cohort, and (right) iRNA expression calculated in an independent FFPE (GSE117439) cohort. Samples are colored according to their estrogen receptor (ER) status. c) Cluster dendrograms from hierarchical boostrap clustering (1000 repetitions) performed with BRCA RNA expression, BRCA iRNA expression, and iRNA expression from the fresh-frozen GSE84207 cohort, and the FFPE GSE117439 cohort. Samples with a “long id name” are ER negative samples. Approximately unbiased p-values (AU) values are given for each cluster node and clusters with AU>0.9 are highlighted by pink rectangles. d+e) Caleydo StratomeX [40] plots showing the concordance between TCGA BRCA microarray based PAM50 subtypes and RNA (d) or iRNA (e) expression based PAM50 subtypes (confidence=1). f) Scatterplot with regression model performance R2 (in independent validation samples) for the 50 genes that constitutes the PAM50 subtype classifier. Top three genes with the highest centroid value is marked for each PAM50 subtype. g) Kaplan–Meier plot showing the overall survival of AJCC stage I-IV patients from the TCGA BRCA cohort stratified according to microarray-based PAM50 subtypes (left panel), RNA-based PAM50 subtypes with confidence call=1 (middle panel), and iRNA-based PAM subtypes with confidence call=1 (right panel). Significance was evaluated by the log-rank test. In parenthesis is provided the Bonferroni-adjusted P values (two comparisons, i.e., LumA vs. HER2 and LumA vs. Basal). h) Consensus cumulative distrib [file 13148_2021_1000_MOESM6_ESM.pdf]

Supplementary Figure 3

a

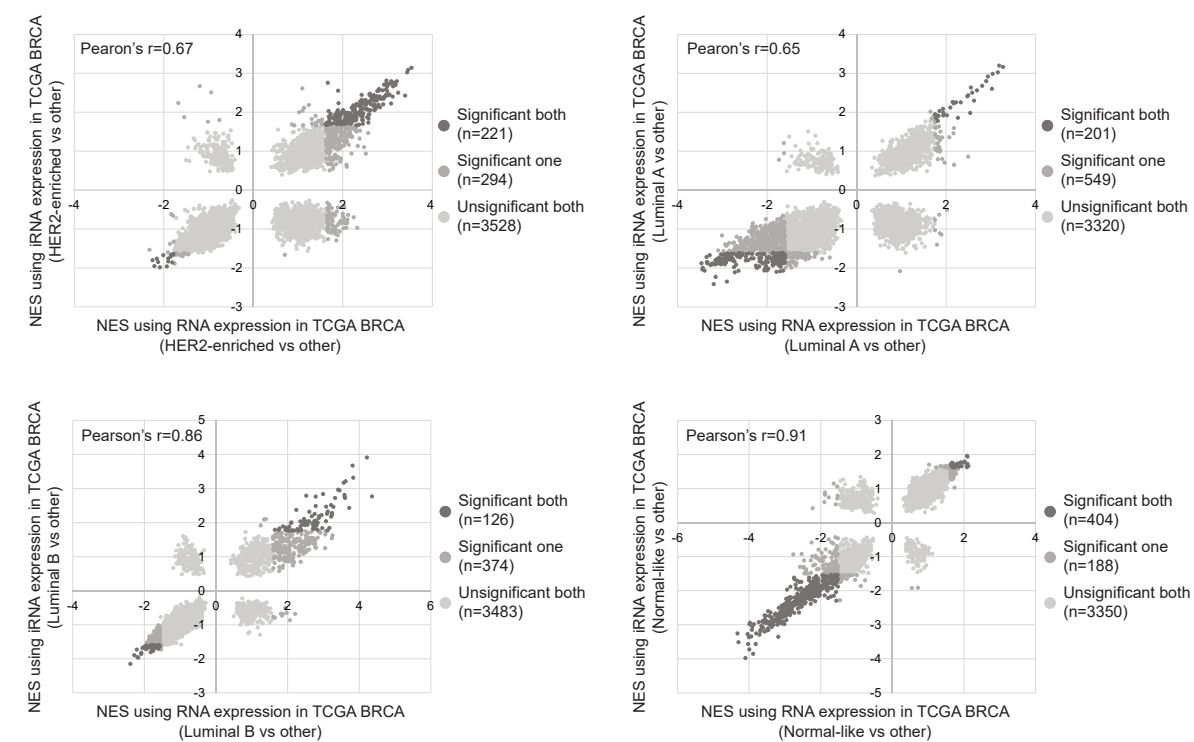

b

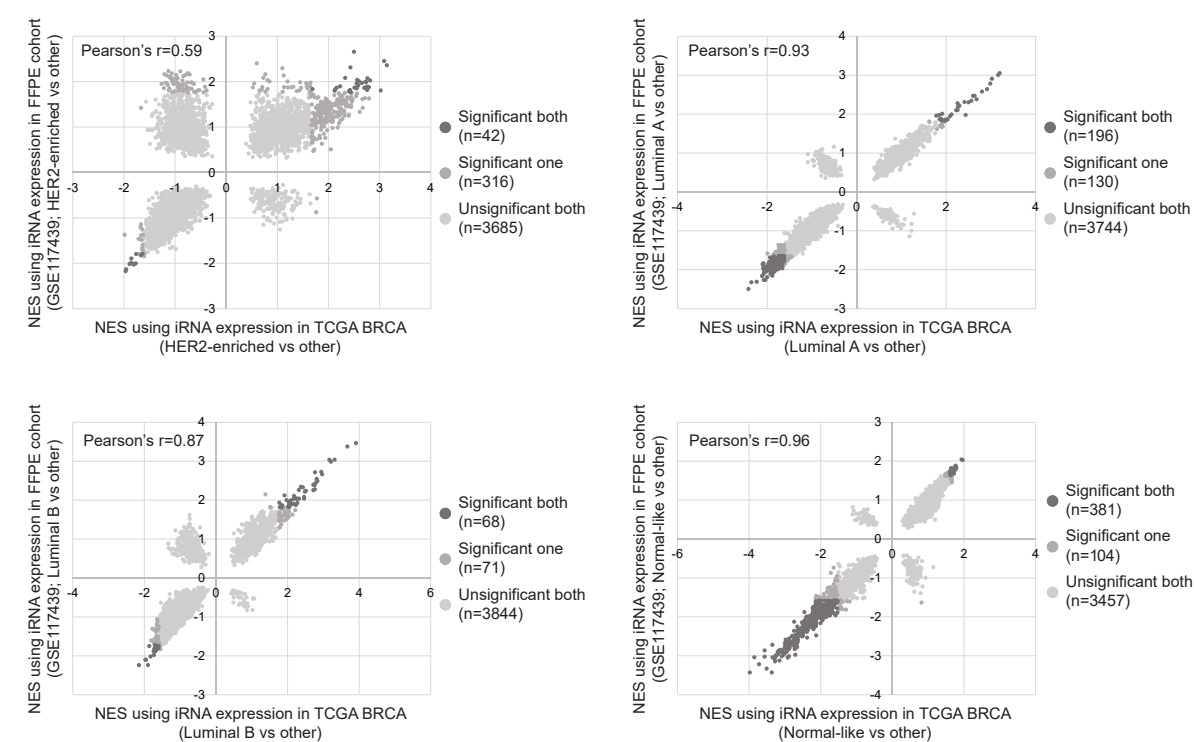

c

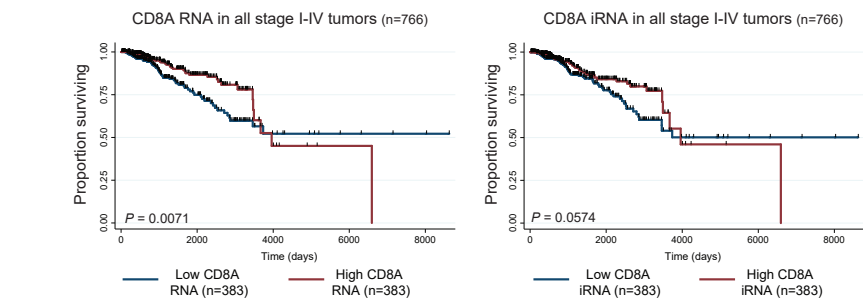

Supplement: Supplementary file 7 — Additional file 7. Figure S3. Subtype characterization with MethCORR inferred RNA expression. a) Scatterplots showing correlations between normalized enrichment sores (NESs) from a gene set enrichment analysis (GSEA) of the TCGA BRCA HER2-enriched subtype vs. all other BRCA samples, the TCGA BRCA Luminal A subtype vs. all other BRCA samples, the TCGA BRCA Luminal B subtype vs. all other BRCA samples, and the TCGA BRCA Normal-like subtype vs all other BRCA samples performed with RNA expression (x-axis) and iRNA expression (y-axis). b) Scatterplots showing correlations between NESs from a GSEA of the HER2-enriched subtype vs. all other samples, the Luminal A subtype vs. all other samples, the Luminal B subtype vs. all other samples, and the Normal-like subtype vs all other samples performed with iRNA expression in the TCGA BRCA cohort (x-axis) and iRNA expression in the independent breast cancer FFPE cohort (GSE117439; y-axis). c) Kaplan–Meier plots showing the overall survival of all AJCC stage I-IV patients from the TCGA BRCA cohort stratified according to high or low CD8A expression (median cut-off) using either RNA (left panel) or iRNA (right panel). Significance was evaluated by the log-rank test. [file 13148_2021_1000_MOESM7_ESM.pdf]
